# The safety of esophagojejunostomy via a transorally inserted-anvil method vs extracorporeal anastomosis using a circular stapler during total gastrectomy for Siewert type 2 adenocarcinoma of the esophagogastric junction

**DOI:** 10.1093/gastro/goz046

**Published:** 2019-10-11

**Authors:** Xin-Hua Chen, Yan-Feng Hu, Jun Luo, Yue-Hong Chen, Hao Liu, Tian Lin, Hao Chen, Guo-Xin Li, Jiang Yu

**Affiliations:** Department of General Surgery, Nanfang Hospital, Southern Medical University, Guangzhou, Guangdong, P. R. China

**Keywords:** adenocarcinoma of the esophagogastric junction, laparoscopic total gastrectomy, esophagojejunostomy, anastomotic leakage

## Abstract

**Background:**

Intracorporeal esophagojejunostomy via a transorally inserted-anvil method during laparoscopic total gastrectomy (LTG) for upper gastric cancer has been demonstrated to be feasible, but the use of this assessment exclusively for Siewert type 2 adenocarcinoma of the esophagogastric junction (AEG) has not been reported.

**Methods:**

A total of 428 consecutive gastric-cancer patients who underwent LTG in Nanfang Hospital from January 2008 to December 2016 were reviewed. Among these patients, 98 were classified as Siewert type 2 AEG. The patients underwent intracorporeal esophagojejunostomy through either a transorally inserted-anvil method (*n *=* *27) or extracorporeal anastomosis usinga circular stapler (*n *=* *71). After generating propensity scores with covariates that were associated with developing anastomotic leakage, 26 patients who underwent esophagojejunostomy via the transorally inserted-anvil method (transoral group) were 1:1 matched with 26 patients who underwent the procedure via extracorporeal anastomosis using a circular stapler (extracorporeal group). The safety after 30 days post-operatively was compared between the two groups.

**Results:**

The transoral group and extracorporeal group were balanced regarding the baseline variables. The operative time, reconstruction duration, number of dissected lymph nodes, length of the proximal resection margins, estimated blood loss, intra-operative complication rate, and post-operative recovery course were not significantly different between the two groups. The mean anvil-insertion completion time (9.7 ± 3.0 vs 13.4 ± 2.0* *minutes, *P *<* *0.001) and the median incision length (5.5 vs 7.0* *cm, *P *<* *0.001) in the transoral group were shorter than those in the extracorporeal group. The incidence of post-operative complications (26.9% vs 23.1%, *P *=* *0.749) and the classification of complication severity (*P *=* *0.939) were similar between the two groups.

**Conclusions:**

Intracorporeal esophagojejunostomy through a transorally inserted-anvil method may be a potentially safe approach to simplify and optimize the procedure during LTG for Siewert type 2 AEG.

## Introduction

Although the incidence of gastric cancer (GC) has declined recently, the proportion of adenocarcinoma of the esophagogastric junction (AEG) has dramatically increased worldwide [1, 2]. Based on the results of the JCOG 9502 trial [3], the abdominal-transhiatal (TH) approach is justified for the treatment of Siewert type 2 AEG. As laparoscopic gastrectomy has been proven to be as safe as open gastrectomy [4–7], the TH approach for AEG is usually performed by laparoscopy.

Conventionally, during laparoscopic total gastrectomy (LTG), esophagojejunostomy is performed through via mini-laparotomy at the upper epigastrium. Nevertheless, due to the limitation of the left inferior phrenic space and the length of the incision, this complicated and challenging procedure becomes more challenging and requires a relatively large incision in order to obtain secure anastomotic procedures, especially for obese patients, thin patients with a high and narrow thoracic oesophagus, or patients who require more extensive resection of the oesophagus [2]. In these situations, the procedure may fail to achieve satisfactory anastomosis and may even easily hurt the liver, spleen, and diaphragm. As a result, some surgeons have turned to thoracotomy, which results in greater trauma accompanied by a higher rate of perioperative morbidity without survival benefits and a loss in the significance of minimally invasive laparoscopy [3, 8, 9]. Thus, many studies of how to perform esophagojejunostomy safely and simply during LTG for upper gastric tumours have been performed [10–15]. However, it is worth noting that the difficulty of LTG for Siewert type 2 AEG is distinguished from that for upper GC because the procedure has to be completed in a high and narrow operation plane.

Excitingly, the transorally inserted-anvil method using OrVil^TM^ can change the orientation of the inserted anvil, simplifying the reconstruction process [16]. Furthermore, when more extensive resection of the oesophagus is required, the transorally inserted-anvil method can reduce the difficulty of esophagojejunostomy and achieve a higher and safer resection margin compared with liner anastomosis [17]. Thus, cases of Siewert type 2 AEG might represent a distinctive treatment indication for the transorally inserted-anvil method. Unfortunately, the exclusive assessment of the procedure for Siewert type 2 AEG has been substantially lacking. In this study, we evaluated the surgical safety and feasibility of intracorporeal esophagojejunostomy through the transorally inserted-anvil method by comparing it with the extracorporeal anastomosis approach during LTG exclusively for Siewert type 2 AEG patients.

## Patients and methods

### Patients

A total of 428 consecutive GC patients underwent LTG at Nanfang Hospital from January 2008 to December 2016. After two independent surgical oncologists retrospectively reviewed the medical records of these patients, it was determined that 98 patients had Siewert type 2 AEG according to the Siewert classification [18]. These patients underwent intracorporeal oesophagojejunostomy via the transorally inserted-anvil method (*n *=* *27) or extracorporeal anastomosis using a circular stapler (*n *=* *71) depending on the patient's choice. Before surgery, the patient was presented with a sufficient explanation about the cost and the characteristics of the different reconstruction approaches. There was no difference between the two procedures in terms of the post-operative care. All patients were treated according to the routine post-operative management protocol used in our department. Patients’ demographics, comorbidities, post-operative results, post-operative recovery courses, and pathologic characteristics were retrospectively analysed based on a prospectively maintained database [19].

**Figure 1.**
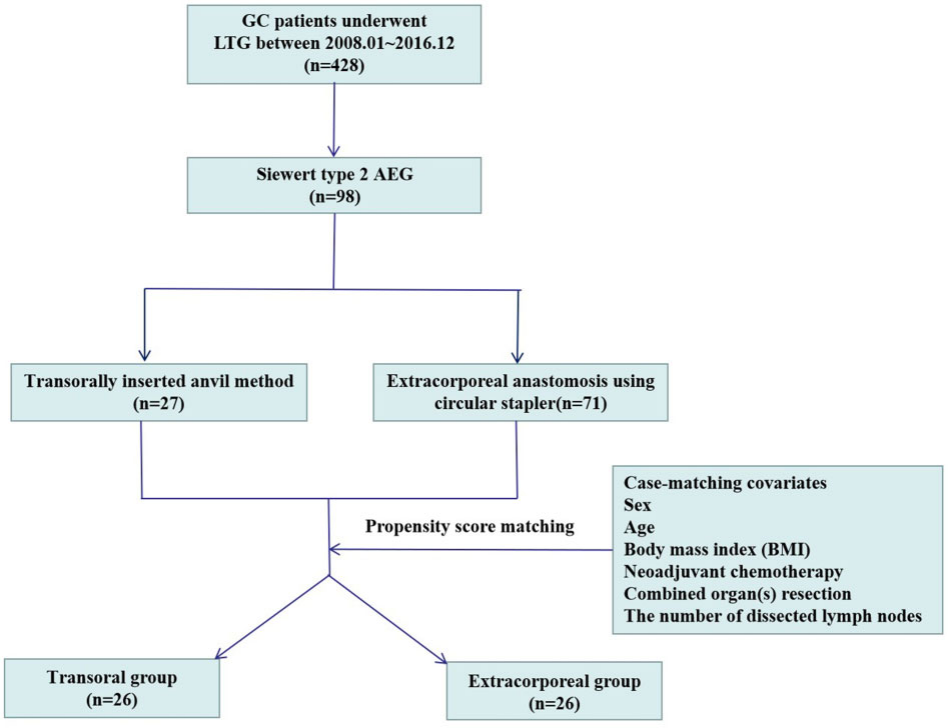
Selection and grouping of stomach-predominant Siewert type 2 adenocarcinoma of esophagogastric junction patients.

After generating propensity score matching (PSM) with six covariates (sex, age, body mass index [BMI], neoadjuvant chemotherapy, combined organ(s) resection, and the number of dissected lymph nodes), 26 Siewert type 2 AEG patients who underwent intracorporeal esophagojejunostomy via the transorally inserted-anvil method (transoral group) and 26 patients who underwent the extracorporeal anastomosis approach (extracorporeal group) were matched with a 1:1 ratio (Figure 1). The post-operative recovery course and safety after 30 days post-operatively were then compared between the two groups. All the Siewert type 2 AEG patients in this trial had stomach-predominant cancer [20] and their staging was determined according to the 7^th^ UICC. This study was approved by the Ethics Committee of Nanfang Hospital, Southern Medical University.

### Surgical procedures

Each patient was placed in the supine position with the legs oriented slightly apart (relaxed dorsal lithotomy position). Dissection of the regional lymph nodes and resection of the stomach were performed as described in our previous study for both approaches [21]. Total gastrectomy with D2 or D2 + No. 10 lymphadenectomy was performed in all patients. Either splenectomy or pancreas- and spleen-preserving splenic hilar lymph-node dissection was conducted to dissect the No. 10 lymph node. The latter was performed as we reported previously [22]. Then, the duodenal bulb was transected using a linear stapler, followed by the transection of the distal oesophagus a proper distance away from the margin of the tumour with the linear stapler.

#### Transorally inserted-anvil method

**Figure 2.**
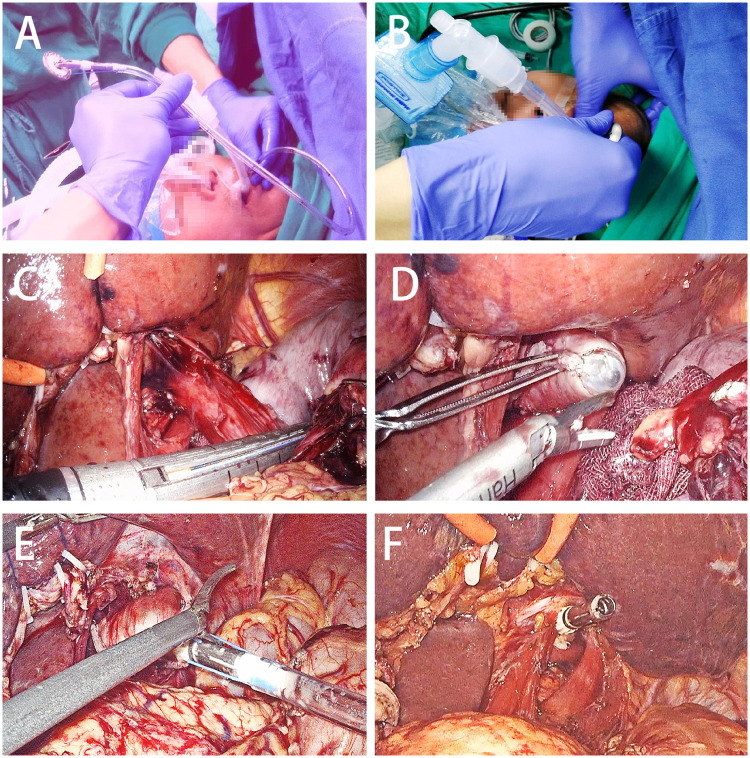
The key course of anvil insertion using the DST series^TM^ EEA^TM^ OrVil^TM^. (A) and (B) OrVil^TM^ anvil was inserted transorally by anaesthetists. (C) The distal oesophagus was transected using a linear stapler. (D) A small cavity was made at the left edge of the staple line in the esophageal stump with a harmonic scalpel. (E) and (F) After the central rod of the anvil was extracted and completely exposed, the tube was dislinked from the anvil by cutting the connecting thread and then taking it away from the abdomen.

After completing the lymph-node dissection, the OrVil^TM^ anvil was inserted transorally by anaesthetists until the tip of the transoral tube reached the position to be transected (Figure 2A and B). After the transection of the distal oesophagus (Figure 2C), the entire specimen was removed and placed into a specimen-collection bag. Concurrently, a small cavity was made on the left edge of the staple line in the oesophageal stump with a harmonic scalpel. Then, the anvil was dragged from the hole using a laparoscopic grasper until the centre rod of the anvil came into view (Figure 2D). After that, the thread connecting the transoral tube and the anvil was cut for release (Figure 2E); then, the tube was removed from the abdominal cavity through one trocar hole. At this point, the insertion of the anvil was complete (Figure 2F).

**Figure 3.**
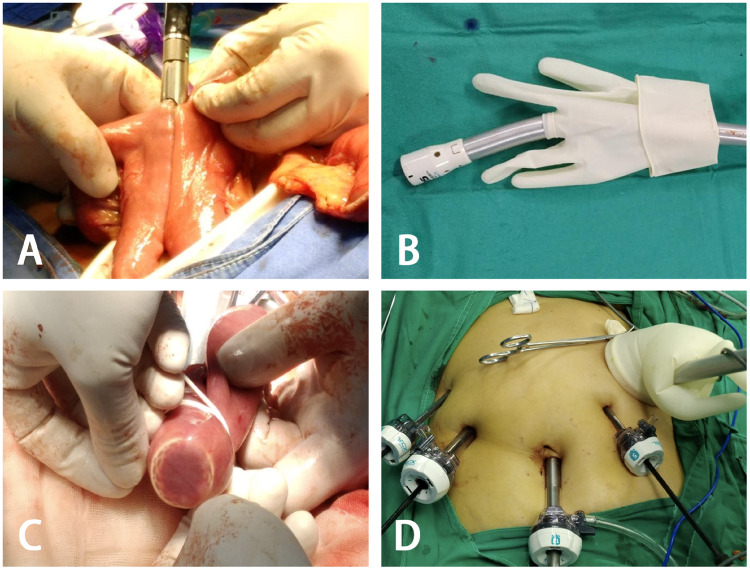
Side-to-side jejunojejunostomy and re-establishment of pneumoperitoneum to prepare for subsequent intracorporeal oesophagojejunal anastomosis. (A) A side-to-side jejunojejunostomy was performed by a linear stapler. (B) Inserting the circular stapler into a surgical glove. (C) A circular stapler was inserted into the jejunal limb and the jejunum was blinded by a rubber band. (D) Sealing off the laparotomy by a self-made single-site access system: a surgical glove attached to a circular stapler and a wound protector.

Later, the left upper port site was extended to 4–6* *cm in length, through which the specimen-collection bag was removed. The jejunum was transected 15* *cm away from the Treitz ligament with the linear stapler, and a side-to-side jejunojejunostomy was then performed with the linear stapler (Figure 3A). The circular stapler was inserted into a surgical glove for subsequent pneumoperitoneum re-establishment (Figure 3B). After that, the circular stapler was positioned within the jejunal limb through the distal jejunal stump. Both jejunal loops on the stapler shaft and the centre rod were anchored with a rubber band to create a special slippage for preventing their separation and tearing the embedded tissues (Figure 3C). Then, the stapler shaft, a surgical glove, and a wound protector were connected as a single-site access system to re-establish the pneumoperitoneum (Figure 3D) and to complete the subsequent intracorporeal oesophagojejunal anastomosis.

**Figure 4.**
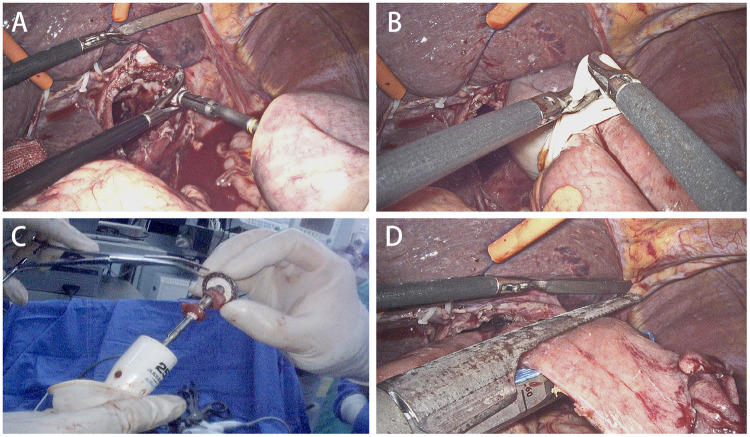
Intracorporeal esophagojejunostomy. (A) The circular stapler and the anvil in the oesophageal stump were linked and fired. (B) Cutting off the rubber band. (C) Taking out the circular stapler and checking the incisal margin after completion of esophagojejunostomy. (D) The jejunal stump was shut down with a linear stapler.

**Figure 5.**
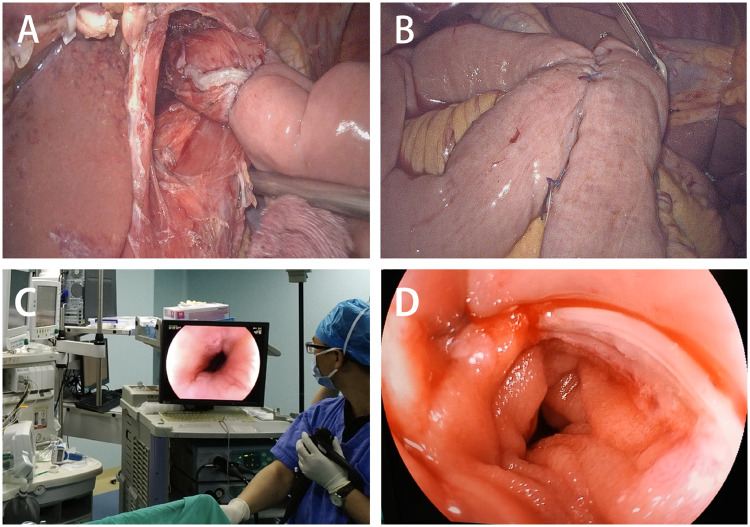
Evaluating the anastomosis routinely. (A) and (B) Evaluation of anastomotic stoma in the laparoscopic view. (C) and (D) Evaluation of anastomotic stoma with on-table endoscopy.

In the laparoscopic view, the circular stapler and the anvil were linked and then fired to complete the anastomosis (Figure 4A). After that, the rubber band was cut off and removed (Figure 4B). The circular stapler was then removed and the incisal margin was checked (Figure 4C). Then, the jejunal stump was closed using the linear stapler (Figure 4D). Last but not least, we assessed the anastomosis by laparoscopy combined with on-table endoscopy (Figure 5).

#### Extracorporeal anastomosis using the circular stapler

The completion of regional lymph-node dissection was followed by making a small midline incision of 8–10* *cm at the epigastrium. Before transecting the distal oesophagus, the purse-string forceps were prepositioned and the suturing was completed. After transecting the distal oesophagus, the anvil was inserted into the proximal oesophagus and the purse-string suture was tightened to represent the anastomosis end of the oesophagus. The specimen was removed via mini-laparotomy. Then, extracorporeal esophagojejunostomy was performed with the circular stapler.

## Outcomes

The primary outcomes ware the rate of early post-operative complications and the anastomotic leakage (AL). Early post-operative complications were assessed within 30 days after the surgery and scaled according to the Clavien–Dindo Classification and Accordion Classification [23], which mainly includes anastomotic complications (leakage, bleeding, and stenosis), intra-abdominal infections, intra-abdominal bleeding, intestinal obstructions, pancreatic fistula, pancreatitis, and wound infections, as well as systemic complications (pulmonary, urinary, renal, hepatic, cardiac, and endocrine). AL was defined as ‘a complete intestinal wall defect at the anastomotic suture line detected with a radiologic contrast medium study or positive color test’ [24]. In our centre, the cases were all confirmed with the methylene blue test and a radiology study with a water-soluble contrast medium and/or endoscopy.

The secondary outcomes were intra-operative complications (any complications that occurred intra-operatively), surgical outcomes (number of dissected lymph nodes, combined organ[s] resection, total operative duration, anvil-insertion duration, reconstruction duration, estimated blood loss, incision length, and length of proximal resection margins) and post-operative recovery (time to first ambulation, time to first flatus, time to first liquid resumption, time to first liquid diet, time to first soft diet, and post-operative hospital stay).

### Statistical analysis

The data were analysed using the SPSS statistical software program (SPSS 22.0). Six covariates (sex, age, BMI, neoadjuvant chemotherapy history, combined organ[s] resection and the number of dissected lymph nodes) were selected to perform PSM. A propensity score was calculated using a logistic-regression model and a nearest-neighbour-matching algorithm. After PSM, the transoral group was 1:1 matched with the extracorporeal group. Data are presented as the mean* *±* *standard deviation for continuous variables (for those with non-normal distributions, medians and ranges are shown) and as numbers (%) for categorical variables. Student *t*-test or the Mann–Whitney U test was used to compare the baseline characteristics and short-term outcomes for continuous variables, while, for categorical variables, the chi-square test or Fisher’s exact test was used. A *P *<* *0.05 was considered statistically significant.

## Results

### Patient characteristics

After PSM, no significant difference was found in age, sex, BMI, neoadjuvant chemotherapy, combined organ(s) resection, or the number of dissected lymph nodes. Both groups were balanced regarding the baseline variables (Table 1).

**Table 1.** Comparisons of baseline characteristics between the two groups.

Variable	Transoral group (*n* = 26)	Extracorporeal group (*n* = 26)	*P*-value
Age, years	61.8 ± 9.1	61.3 ± 7.9	0.834
Gender			1.000
Male	22 (84.6)	21 (80.8)	
Female	4 (15.4)	5 (19.2)	
Body mass index, kg/m²	22.5 ± 3.4	21.6 ± 3.3	0.353
Hemoglobin, g/L	103.2 ± 35.3	108.4 ± 36.4	0.610
Albumin, g/L	37.0 ± 4.0	38.7 ± 3.8	0.145
ECOG score, *n* (%)			0.272
0	13 (50.0)	15 (57.7)	
1	7 (26.9)	10 (38.5)	
2	3 (11.5)	1 (3.8)	
3	3 (11.5)	0 (0.0)	
Comorbidities			
Hypertension	5 (19.2)	4 (15.4)	1.000
Diabetes mellitus	2 (7.7)	3 (11.5)	1.000
Cardiovascular disease	0 (0.0)	2 (7.7)	0.471
Pulmonary disease	2 (7.7)	1 (3.8)	1.000
Hepatic disease	1 (3.8)	5 (19.2)	0.193
History of abdominal surgery	0 (0.0)	2 (7.7)	0.471
Neoadjuvant chemotherapy	2 (7.7)	5 (19.2)	0.416
Tumour diameter, cm	46.0 ± 14.1	47.1 ± 14.1	0.791
TMN stage			0.569
I	0 (0.0)	1 (3.8)	
II	7 (26.9)	10 (38.5)	
III	19 (73.1)	12 (46.2)	
IV	0 (0.0)	3 (11.5)	

Values presented with mean ± standard deviation or *n* (%). ECOG, Eastern Cooperative Oncology Group.

### Surgical outcomes

The total operative time and duration of reconstruction were not significantly different between the two groups (both *P *>* *0.05), whereas the anvil-insertion completion time was shorter in the transoral group than in the extracorporeal group (9.7* *±* *3.0 vs 13.4 ± 2.12 minutes, *P *<* *0.001). The median length of the incision was shorter in the transoral group than in the extracorporeal group (5.5 vs 7.0* *cm, *P *<* *0.001). The length of the proximal resection margins, estimated blood loss, number of retrieved lymph nodes, intra-operative complication rate, and post-operative recovery course (including the time to first ambulation, flatus, liquid resumption, liquid diet, soft diet, and post-operative hospital stay) showed no significant difference (Table 2).

**Table 2.** Comparisons of surgical outcomes between the two groups

Variable	Transoral group (*n* = 26)	Extracorporeal group (*n* = 26)	*P*-value
Number of dissected lymph nodes	40.1 ± 18.3	30.8 ± 18.8	0.163
Combined organ(s) resection	1 (3.8)	3 (11.5)	0.603
Total operative duration, minutes	234.8 ± 33.9	227.4 ± 38.1	0.457
Anvil insertion^a^	9.7 ± 3.0	13.4 ± 2.2	<0.001
Reconstruction	48.4 ± 12.6	53.9 ± 8.3	0.067
Estimated blood loss, mL	124.2 ± 115.4	143.5 ± 130.6	0.576
Incision length, cm	5.5 [1.0]	7.0 [2.0]	<0.001
Length of proximal resection margins, mm	25.4 ± 16.2	27.3 ± 15.5	0.666
Time to first ambulation, days	3.6 ± 3.9	4.9 ± 5.9	0.348
Time to first flatus, days	3.0 [1.0]	3.0 [1.0]	0.116
Time to first liquid resumption, days	3.2 ± 1.2	4.7 ± 3.9	0.075
Time to first liquid diet, days	4.4 ± 1.3	5.9 ± 4.1	0.086
Time to first soft diet, days	6.2 ± 2.0	7.6 ± 4.6	0.170
Post-operative hospital stay, days	14.4 ± 18.4	16.2 ± 20.3	0.743

Values presented with mean ± standard deviation, median [interquartile range], or *n* (%).

^a^ Anvil-insertion time in the transoral group: from transection of the oesophagus to disconnection of the thread linking the transoral tube and the anvil; anvil-insertion time in the extracorporeal group: from preposition of the purse-string forceps to completion of anvil fixation.

**Table 3.** Comparisons of perioperative complications between the two groups

Variable	Transoral group (*n* = 26)	Extracorporeal group (*n* = 26)	*P*-value
Intra-operative complications, *n* (%)	3 (11.5)	2 (7.7)	1.000
Spleen injury	2 (7.7)	1 (3.8)	1.000
Vessel bleeding	1 (3.8)	1 (3.8)	1.000
Post-operative complications, *n* (%)	7 (26.9)	6 (23.1)	0.749
Anastomotic complications	3 (11.5)	4 (15.4)	1.000
Anastomotic leakage	3 (11.5)	3 (11.5)	1.000
Anastomotic bleeding	0 (0.0)	2 (7.7)	0.471
Intra-abdominal infection	2 (7.7)	3 (11.5)	1.000
Mediastinal infection	0 (0.0)	1 (3.8)	1.000
Ileus	0 (0.0)	3 (11.5)	0.234
Wound infection	0 (0.0)	1 (3.8)	1.000
Pneumonia	6 (23.1)	5 (19.2)	1.000
Pancreatic fistula	1(3.8)	1 (3.8)	1.000
Liver dysfunction	0 (0.0)	1 (3.8)	1.000
Complication classification^a^, *n* (%)			0.939
I	0 (0.0)	1 (3.8)	
II	4 (15.4)	2 (7.7)	
IIIa	1 (3.8)	1 (3.8)	
IV	2 (7.7)	2 (7.7)	
Second operation, *n* (%)	2 (7.7)	2 (7.7)	1.000

^a^ According to the Clavien–Dindo classification.

### Intra-operative and post-operative complications

The intra-operative complications, including spleen injury and vessel bleeding, were similar between the two groups (11.5% vs 7.7%, *P *=* *1.000). The incidence of post-operative complications (26.9% vs 23.1%, *P *=* *0.749) and the classification of complication severity (*P *=* *0.939) were approximately the same. Notably, the rates of anastomotic complications and anastomotic bleeding seemed to be lower in the transoral group than those in the extracorporeal group, but the difference was not significant (11.5% vs 19.2%, *P *=* *0.702 and 0.0% vs 7.7%, *P *=* *0.471, respectively). AL was 11.5% in both groups and one of the cases of AL also demonstrated anastomotic bleeding in the extracorporeal group. Two-thirds of the patients with AL required a second operation in both groups. In addition, the rate of pancreatic fistula was 3.85% in both groups (Table 3).

The most common post-operative complication was pneumonia in both groups (23.1% vs 19.2%, *P *=* *1.000). In the transoral group, three out of six cases of pneumonia followed AL and/or pancreatic fistula, while, in the extracorporeal group, all five cases of pneumonia were accompanied by anastomotic complications and/or pancreatic fistula. In addition, there was no significant difference between the two groups regarding intra-abdominal infection (*P *=* *1.000), ileus (*P *=* *0.234), mediastinal infection (*P *=* *1.000), wound infection (*P *=* *1.000), or liver dysfunction (*P *=* *1.000). In addition, all these post-operative complications were mainly attributed to the occurrence of AL and pancreatic fistula, except for three cases of pneumonia in the transoral group and one case of liver dysfunction in the extracorporeal group.

## Discussion

Esophagojejunostomy during LTG for Siewert type 2 AEG remains a challenge and more studies are needed to explore an optimal method. Based on the results of the trials concerning the feasibility of esophagojejunostomy during LTG for upper gastric tumours, the transorally inserted-anvil method has the advantage of changing the direction of anvil insertion, simplifying the reconstruction process and decreasing the duration of esophagojejunostomy [25–29]. Thus, in this trial, we aimed to evaluate the surgical safety and feasibility of intracorporeal esophagojejunostomy through the transorally inserted-anvil method during LTG for Siewert type 2 AEG.

Reviewing the trials concerning the feasibility of the transorally inserted-anvil method for esophagojejunostomy during LTG for upper gastric tumours, we found some limitations and learned from them to obtain comparable and reliable evidence. Most of the previous studies focused on introducing their experience with a few cases [30–32]. Some were significantly heterogeneous in terms of the number of cases included. A study conducted by Chong-Wei *et al*. [33] enrolled patients whose primary diseases differed from Zollinger–Ellison syndrome, with diseases ranging from stromal tumours in the cardia to adenocarcinoma in the stomach. The patients included in a study carried out by Marangoni *et al.* [25] underwent different surgeries, including laparoscopic Ivor–Lewis esophagectomy, total gastrectomy, and subtotal gastrectomy. Choi *et al.* [34] studied the incidence rate of AL and stricture of esophagojejunostomy with the transorally inserted-anvil method. However, the operative approaches in the study included open (51.7%), laparoscopic (43.3%), and robotic (5.0%) approaches, and the range of gastric-resection approaches included total gastrectomy (81.7%), proximal gastrectomy (10.0%), and completion gastrectomy (8.3%). Jung *et al.* [26] compared the safety of intracorporeal circular stapling esophagojejunostomy via the transorally inserted-anvil method with the extracorporeal anastomosis approach. The time period in the transoral group was from 2009 to 2014, while that in the extracorporeal group was between 2004 and 2008. As far as we know, the treatment types and nursing methods used for GC differ in these two separate periods. Shida *et al.* [27] assessed the usefulness and safety of esophagojejunostomy through the transorally inserted-anvil method by analysing GC patients with tumour locations in the upper third, middle third, upper to middle, and lower to upper regions, and the surgeries included both LTG and open total gastrectomy. Few previous studies have investigated the feasibility and advantages of esophagojejunostomy with the transorally inserted-anvil method during LTG, focusing exclusively on Siewert type 2 AEG patients.

Moreover, some related studies have compared the transorally inserted-anvil method with another anastomosis approach with selection bias and differences in baseline characteristics [28, 29]. These limitations may impair the objectivity of the results. PSM was conducted to compensate for selection bias and to avoid potential confounding effects. Thus, in this study, we chose consecutive Siewert type 2 AEG patients in the same period and performed a PSM analysis to further balance the factors that may affect the assessment of the safety of anastomosis, not only the baseline variables that were unbalanced between the two groups. The risk factors for the development of AL in patients undergoing LTG according to the data of our centre include sex, age, neoadjuvant chemotherapy, combined organ(s) resection, and invasion of the oesophagus [35]. After PSM, the baseline and treatment-related characteristics were balanced and patients were 1:1 matched between the two approaches.

Insecure anastomosis may cause severe complications, especially AL, which will prolong the post-operative hospital stay, increase medical costs, and even affect long-term prognosis [36, 37]. According to the nationwide internet-based database of Japan, the incidence of AL after total gastrectomy was 4.4% (881/20011) in 2011 [38]. The systematic analysis reported a similar incidence of 2.45% [39]. Therefore, AL was regarded as one of the most critical post-operative complications clinically and the incidence of AL has been deemed one of the key quality indicators to investigate the safety of esophagojejunostomy approaches after total gastrectomy in this study. In the present study, the incidence rate of AL reached 11.5%, which is quite high compared with previous reports about the safety of the transorally inserted-anvil method for esophagojejunostomy during LTG. However, in fact, the data for our centre indicated that the incidence rate of AL after total gastrectomy was 2.3% (12/525) from January 2008 to December 2016. The difference occurred because the factors used to perform PSM were risk factors for the development of AL according to the data in our centre [39]. Additionally, these factors in both groups after PSM were at a high level. Among the six Siewert type 2 AEG patients with AL, all were male with a mean age of 65.5 years, two received neoadjuvant chemotherapy, and one received chemotherapy combined with organ resection. Thus, the high incidence rate of AL in the Siewert type 2 AEG patients in this study was reasonable.

Interestingly, contrary to the results reported by Kawamura *et al.* [40], which showed that the esophagojejunostomy procedure via the transorally inserted-anvil method was an independent risk factor for anastomotic complications during LTG, our finding indicated that esophagojejunostomy via the transorally inserted-anvil method during LTG for Siewert type 2 AEG patients achieved a lower risk of anastomotic complications, although the difference was not significant. Furthermore, the transorally inserted-anvil method can simplify the esophagojejunostomy procedure and take advantage of the duration of anvil insertion and the mean length of incision without impairing its safety. The development of LTG has been limited mainly because of the difficulty of reconstruction of the digestive tract, especially with esophagojejunostomy, which is seen as the most complicated and difficult part of the procedure even by experienced surgeons. Hence, the short time of anvil insertion and small incision reflect the simplification and optimization of the surgical procedure to a large extent. Therefore, explorations of how to perform oesophagojejunostomy safely and simply during LTG in Siewert type 2 AEG patients should take into account the transorally inserted-anvil method.

The limitations of our study are also apparent. Although the data in our study were prospectively collected, our study was retrospectively analysed and the inherent selection bias was adjusted but not completely eliminated by using PSM. Furthermore, since the cardinal number of Siewert type 2 AEG patients was limited, the number of samples in the analysis was small, which may have impaired the power of the test. In addition, the fact that esophagojejunostomy with the extracorporeal anastomosis approach is conventional and performed proficiently in our centre may weaken the advantage of the transorally inserted-anvil method in the study. Last but not least, because the follow-up was routinely conducted and included a complete detailed assessment of the anastomotic stoma 1 month after surgery, the recording of anastomotic stenosis was not complete and the occurrence of anastomotic stenosis might have been lower than the actual condition. As a result, we abandoned the analysis of anastomotic stenosis. Thus, the assessment of anastomotic stenosis needs to be prospectively investigated in a well-designed randomized trial.

In conclusion, intracorporeal esophagojejunostomy via a transorally inserted-anvil method may be a potentially safe approach to simplify and optimize the esophagojejunostomy procedure during LTG in Siewert type 2 AEG patients.

## Funding

This work was supported by Medtronic, National Key Clinical Specialities Construction Program of China [No. [2012]121], Science and Technology Planning Project of Guangdong Province [2013B021800313], and Special Funds for the Cultivation of Guangdong College Students' Scientific and Technological Innovation [pdjha0094].

## Authors’ contributions

Study conception and design: G.X.L., J.Y. Acquisition of data: X.H.C., Y.F.H., J.L., Y.H.C., H.L., T.L., H.C. Data analysis and interpretation: X.H.C., Y.F.H., J.L., Y.H.C., H.L., T.L., H.C. Drafting of manuscript: X.C., Y.F.H. Critical revision: G.X.L., J.Y. All authors read and approved the final version.
